# The Yeast in the Vial: Iatrogenic *Candida viswanathii* Infection Associated with Contaminated Milrinone

**DOI:** 10.3390/jof12090646

**Published:** 2026-09-01

**Authors:** Terenzio Cosio, Elena De Carolis, Nadia Mores, Riccardo Torelli, Brunella Posteraro, Giuseppe Vetrugno, Tiziana D’Inzeo, Giovanni Vento, Maurizio Sanguinetti

**Affiliations:** 1 Dipartimento di Scienze di Laboratorio ed Ematologiche, Fondazione Policlinico Universitario A. Gemelli IRCCS, 00186 Rome, Italy; riccardo.torelli@policlinicogemelli.it (R.T.); brunella.posteraro@unicatt.it (B.P.); tiziana.dinzeo@unicatt.it (T.D.); maurizio.sanguinetti@unicatt.it (M.S.); 2 Institute of Pharmacology, Pharmacovigilance, Policlinico Universitario A. Gemelli, 00186 Rome, Italy; nadia.mores@policlinicogemelli.it; 3 Department of Health Surveillance and Bioethics, Section of Legal Medicine, Fondazione Policlinico Universitario A. Gemelli IRCCS, 00186 Rome, Italy; giuseppe.vetrugno@unicatt.it; 4 Neonatology Unit, Department of Woman and Child Health and Public Health, Fondazione Policlinico Universitario ‘Agostino Gemelli’ IRCCS, 00186 Rome, Italy; giovanni.vento@policlinicogemelli.it

**Keywords:** *Candida viswanathii*, candidemia, case report, iatrogenic infection, milrinone, contaminated medicinal product, extremely preterm infant, pharmacovigilance

## Abstract

Background: Iatrogenic fungal infections associated with contaminated parenteral products are uncommon but potentially severe, particularly when the affected patients are critically ill and the microorganism is not included in routine diagnostic panels. Here, we describe *C. viswanathii* candidemia associated with contaminated milrinone in an extremely preterm infant and place the case within the context of the published clinical evidence. Methods: The infant’s clinical course, microbiological findings, milrinone exposure and pharmaceutical-source investigation were retrospectively reconstructed. Published human *C. viswanathii* infections were reviewed systematically, together with Italian pharmacovigilance communications concerning milrinone. Results: A 600 g preterm infant developed candidemia on Day 7 of life. A central-venous-catheter (CVC) blood culture became positive for yeasts after 7 h, while the BIOFIRE^®^ Blood Culture Identification 2 (BCID2) Panel remained negative. *C. viswanathii* was recovered from blood, urine and bronchoalveolar lavage fluid (BAL). Voriconazole was started, and anidulafungin was subsequently added. The infant died on Day 13 following accidental extubation and unsuccessful resuscitation. Exposure reconstruction identified a contaminated milrinone lot, and the source investigation linked the case to the wider Italian multicenter cluster. The literature review showed that *C. viswanathii* can cause bloodstream, central nervous system, deep-seated and osteoarticular infection, with variable susceptibility, particularly for azoles, but no previous iatrogenic sources were found, and its ecological niche remains undefined. Conclusion: This case provides additional evidence that the Italian *C. viswanathii* outbreak was an iatrogenic event associated with contaminated milrinone. Recognition depended on combining extended yeast identification with medication-lot traceability and interinstitutional pharmacovigilance.

## 1. Introduction

Iatrogenic fungal infections, arising directly from healthcare interventions such as the use of contaminated medicinal products, invasive procedures, indwelling devices that breach mucocutaneous barriers, or other lapses in asepsis, constitute a distinct subset of healthcare-associated infections in which the causal chain is attributable to the intervention itself [1,2]. These infections are a recurrent yet under-recognized threat, capable of causing severe morbidity, mortality and large-scale outbreaks [1,2,3,4]. Although often framed as rare mishaps, iatrogenic fungal infections have recurred over the past few decades and have produced some of the most severe healthcare-associated outbreaks described to date [1,2,3,4,5,6,7,8,9,10,11,12,13,14,15,16,17,18,19,20]. Historical episodes involving *Exserohilum rostratum* in contaminated corticosteroid preparations, *Candida parapsilosis* in total parenteral nutrition (TPN), and *Saccharomyces cerevisiae* and *Malassezia pachydermatis* in probiotic and lipid formulations have shown how exogenous fungal contamination can traverse quality-control barriers and reach highly vulnerable patients [5,6,16,19,20].

These episodes, spanning different continents, patient populations and fungal species, converge on a shared set of structural weaknesses: the ability of nutrient-rich or lipid-containing formulations to support microbial persistence or growth, contamination introduced during manufacture, compounding or clinical handling, and distribution networks capable of disseminating a single quality failure across institutions and countries [13,14,15,16,17,18,19,20]. Despite these experiences, fungal contamination of medicinal products is still largely approached as an exceptional breach. Quality control tends to focus on bacteria and selected indicator organisms; routine environmental monitoring and product testing rarely prioritize fungi, and pharmacovigilance systems are not designed to detect rare yeasts with limited phenotypic distinction [4,21]. Regulatory frameworks such as the Food and Drug Administration (FDA) current good manufacturing practice (CGMP) guidance on aseptic processing and the European Union GMP and sterilization guidelines provide general requirements to prevent microbial contamination, including fungi [22,23]. However, only after the 2012 fungal meningitis outbreak were more targeted measures introduced in the United States (US), such as the Drug Quality and Security Act and FDA guidance on insanitary conditions at compounding facilities, explicitly acknowledging the risk of fungal contamination of injectable products [24]. Together, these events demonstrate that fungal contamination of medicinal products is a systemic vulnerability of the international pharmaceutical supply chain. In most documented outbreaks, contamination occurred at the production or compounding site, and frequently in a different country from where infections emerged, allowing fungi to cross borders via the global distribution of intravenous and parenteral preparations [25,26]. *Candida viswanathii* epitomizes this dual vulnerability.

Its initial recovery from two patients with meningitis in India, first in 1959 and subsequently in the 1970s, was regarded as an exceptional clinical finding [27,28]. Later reports from different countries and anatomical sites established its ability to cause both localized and invasive disease [27,28,29,30,31,32,33,34]. It has been recovered sporadically from soil, petroleum-contaminated terrestrial and marine environments, and animal-associated samples, although its ecological niche and natural reservoir remain undefined [35,36,37,38]. More recently, the occurrence of hospital clusters has revealed its potential as a healthcare-associated pathogen. Its increased visibility probably reflects both genuine nosocomial emergence and improved species resolution through molecular sequencing and expanded matrix-assisted laser desorption/ionization time-of-flight mass spectrometry (MALDI-TOF MS) reference libraries [30,31,32,33,34].

In 2025, a pediatric hospital in Italy reported the first recognized European outbreak of *C. viswanathii*, comprising multiple bloodstream isolates initially identified as *Candida tropicalis* and undetected by the BIOFIRE^®^ Blood Culture Identification 2 (BCID2) Panel (FilmArray^®^) [33,34]. In the same period (September–October 2025), we characterized microbiological features of a suspected isolate from a neonatal intensive care unit (NICU) patient to our hospital, including multiplatform MALDI-TOF MS analyses, internal transcribed spacer (ITS) sequencing, Fourier-transform infrared (FTIR) spectroscopy and antifungal susceptibility testing, finally identified as *C. viswanathii* [39]. Subsequent Italian pharmacovigilance investigations (November 2025) associated *C. viswanathii* infections reported at several hospitals, including the previous one at our hospital [35], with milrinone 10 mg/10 mL, an imported milrinone formulation, followed by prompted distribution suspension, active case finding and further quality investigations [34,40,41]. In this context, we describe the clinical course of *C. viswanathii* candidemia in an extremely preterm infant and review the available literature, with particular attention to healthcare-associated acquisition, antifungal management, and the microbiological and epidemiological evidence linking the infection to contaminated milrinone.

## 2. Case Report

**Figure 1:**
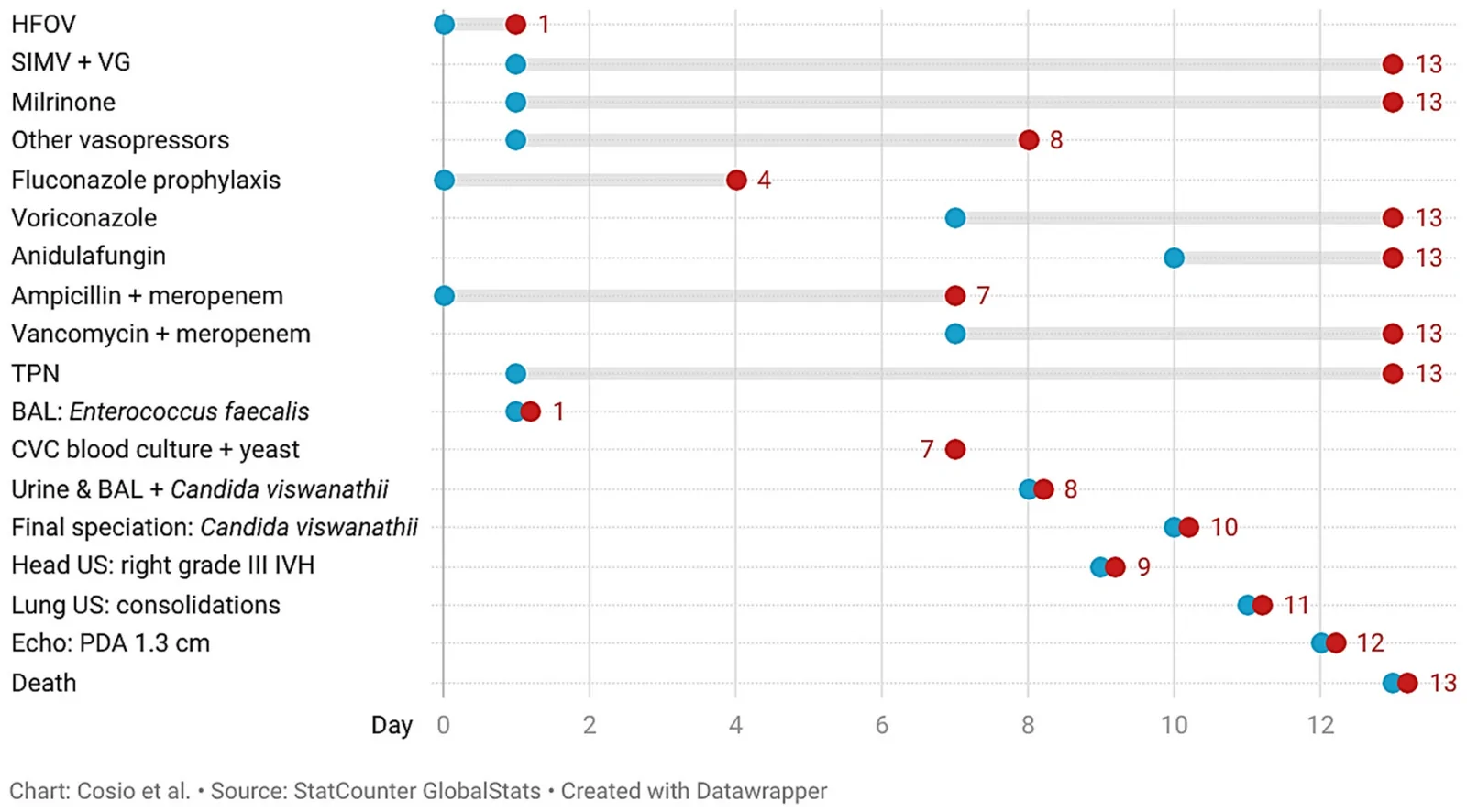
Clinical and microbiological timeline. Timeline of respiratory and cardiovascular support, antimicrobial therapy, parenteral nutrition and key diagnostic events from birth/admission (Day 0 = 22 September 2025) to death (Day 13 = 5 October 2025). Bars indicate the duration of each intervention, while dots mark discrete events. Abbreviations: HFOV, high-frequency oscillatory ventilation; SIMV, synchronized intermittent mandatory ventilation; VG, volume guarantee; TPN, total parenteral nutrition; CVC, central venous catheter; BAL, bronchoalveolar lavage; US, ultrasound; IVH, intraventricular hemorrhage; PDA, patent ductus arteriosus. Blue dot, first day; Red dot, last day. Created with Datawrapped.

A profoundly premature female infant (birth weight 0.60 kg, length 29 cm, and head circumference 21.5 cm) was admitted to the NICU of Fondazione Policlinico Universitario A. Gemelli on 22 September 2025 after prolonged rupture of membranes exceeding 18 h with malodorous vaginal losses and presumed chorioamnionitis. Admission blood gas showed mixed acidosis with pH 7.19, pCO_2_ 49 mmHg and pO_2_ 98 mmHg. Per unit protocol, she received vitamin K 0.2 mg intramuscularly, caffeine citrate 12 mg intravenous (IV) loading followed by 5 mg maintenance, fluconazole prophylaxis 3 mg IV, ophthalmic tobramycin, and empiric ampicillin–meropenem (30 + 12 mg IV). During the first 48 h, she remained critically unstable. On Day 1, progressive mixed acidosis with hyperkaliemia and evolving cardiorespiratory compromise required re-intubation and transition to synchronized intermittent mandatory ventilation with volume guarantee. Vasoactive support (IV milrinone 0.441 mg/24 h and fenoldopam 0.1 µg/kg/min) was initiated and tense ascites was drained with immediate improvement in oxygenation. A bronchoalveolar lavage (BAL) was positive for *Enterococcus faecalis*. Total parenteral nutrition commenced, and she remained *nil per os*. Over Days 2 to 6, she continued invasive ventilation, vasoactive infusions, sedation and analgesia, with persistent mixed respiratory acidosis with pH approximately 7.12 to 7.18 and pCO_2_ 49 to 61 mmHg, mild hyponatremia, transient renal impairment with preserved urine output and rising bilirubin levels. On Day 7, a sepsis work-up was performed for persistent instability. Blood cultures drawn from the central venous catheter (CVC) signaled positive at 7 h, and microscopy demonstrated yeast forms, while the BIOFIRE^®^ FILMARRAY^®^ Blood Culture Identification 2 (BCID2) Panel (BioFire Diagnostics, LLC, Salt Lake City, UT, USA) was negative [39]. Voriconazole 5.7 mg IV was started for presumed catheter-related candidemia, and antibacterial therapy was rationalized to vancomycin and meropenem (10.5 + 14 mg IV). Over subsequent days, she developed progressive multisystem involvement. On Day 9, cranial ultrasound documented a right grade II intraventricular hemorrhage. On Day 10, the suspected source was removed, and a new femoral CVC was inserted under full sterile barrier precautions, with taurolidine locks used around TPN changes. Anidulafungin 2.5 mg IV was added for combination antifungal coverage. The initial blood culture was identified as *C. viswanathii* by three MALDI-TOF MS platforms, with subsequent positivity from urine and repeated BALs, for a total of four *Candida* clinical isolates [39]. The minimum inhibitory concentration (MIC) results (Sensititre YeastOne^®^ ITAMYUCC custom panel; Thermo Fisher Scientific, Oakwood, GA, USA) on the four different clinical isolates of *C. viswanathii* revealed low values across all antifungal classes, except for fluconazole (2–4 µg/mL) (Table S1) [35]. Lung ultrasound on Day 11 revealed multiple consolidations, and echocardiography on Day 12 demonstrated a large pulsatile patent ductus arteriosus measuring 1.3 cm with left-sided volume overload. Despite ongoing ventilatory and vasoactive support, she deteriorated and died on 5 October 2025 after an accidental extubation and unsuccessful resuscitation. No autopsy was performed at the parents’ request. The clinical course is summarized in Figure 1. A national audit in November 2025 then identified the same species in retained vials of milrinone (lot A25138A; expiry 31 January 2027), which was the same lot used in our hospital. In response, the hospital issued an urgent circular mandating complete recall and prohibition of use of this lot, with immediate return of stock. The lot had been manufactured in India, and concordant reports from other hospitals of similar *C. viswanathii* candidemia in patients exposed to milrinone, from the same batch, supported the interpretation of a drug-contamination cluster [33,34].

## 3. Materials and Methods

We performed a systematic literature search of the following databases from 1959 to August 2026: Cochrane Central Register of Controlled Trials; MEDLINE via PubMed; Embase; US National Institutes of Health Ongoing Trials Register; NIHR Clinical Research Network Portfolio Database; and the World Health Organization International Clinical Trials Registry Platform. Reference lists and published systematic review articles were analyzed. We used the terms “*Candida viswanathii*” and/or “*Candida lodderae*” with the following keywords, separately and in combination: “sepsis”, “septicemia”, “bacteremia”, “bloodstream infection”,” candidemia”, “infection”, “outbreak” and “contamination”. Only English-language articles were included in the search. Forward citation analyses of original studies and review articles were also conducted. Where other bacterial or fungal organisms were co-isolated from the same patient, only data pertaining to *C. viswanathii* were extracted. All human studies were included with no age-, sex-, ethnicity-, or type-of-scientific-study-related restriction. We considered only case reports and case series in which *C. viswanathii* was a pathogen causing infections and all case reports that have not yet been included in reviews or clinical trials. Exclusion criteria were articles discussing non-*C. viswanathii* infection and non-English-language publications.

## 4. Results

The literature review included nine publications describing human *C. viswanathii* infections [27,28,29,30,31,32,33,34,42]. The available evidence comprised individual case reports, surveillance data, one clinical series and two complementary investigations of the 2025 Italian outbreak. Given the limited and heterogeneous evidence base, the clinical, epidemiological and microbiological findings were synthesized descriptively in Table 1, while the available antifungal susceptibility data are summarized in Table S2.

Taken together, the published reports suggest that *C. viswanathii* has a broader geographic and clinical distribution than indicated by the earliest descriptions, although the available evidence remains limited to a small number of heterogeneous reports. Beyond the two original Indian meningitis cases [27,28], subsequent reports comprised candidemia detected through surveillance in Argentina [30]; an Indian series of invasive candidiasis involving bloodstream, deep-seated, cerebrospinal fluid and pulmonary specimens [30]; recurrent septic arthritis and osteomyelitis in Singapore and in India [32,42]; and pediatric bloodstream infections during the 2025 Italian outbreak [33,34,39]. The patients range from children and premature neonates to older adults and include both immunocompromised hosts, including patients with prematurity, neutropenia or chronic comorbidities, and immunocompetent individuals who had nevertheless undergone healthcare-related procedures [27,28,29,30,31,32,33,34].

Although the number of tested isolates remains small, the available antifungal susceptibility data show generally low numerical MICs for amphotericin B and echinocandins, whereas azole MICs are more heterogeneous, including markedly elevated fluconazole MICs among a subset of the Indian isolates (Table S2) [31,32,33,39]. When clinical information was available, infections frequently occurred in the setting of host vulnerability, invasive healthcare exposure or disruption of anatomical barriers through surgery, intravascular or intrathecal devices, or intra-articular procedures. These findings support the interpretation of *C. viswanathii* as an opportunistic pathogen capable of causing healthcare-associated invasive infection. Interestingly, the published Indian cases showed a relative concentration in northern India: the first two cerebrospinal fluid isolates were reported from Delhi, whereas the largest clinical series originated from a single tertiary-care center in Chandigarh. No published clinical case included in the review originated from Gujarat, where the implicated milrinone vials were subsequently manufactured.

## 5. Discussion

### 5.1. Case in Context

Although often framed as rare mishaps, iatrogenic fungal infections have recurred over recent decades and have produced some of the most severe healthcare-associated outbreaks described to date [1,2,3,4,5,6,7,8,9,10,11,12,13,14,15,16,17,18,19,20,21]. The limited published literature documents *C. viswanathii* infections involving the bloodstream, central nervous system, deep-seated sites and the osteoarticular system across heterogeneous clinical contexts [27,28,29,30,31,32,33,34,42]. However, the scarcity and heterogeneity of the available reports preclude determining whether the species is associated with a characteristic clinical syndrome or specific tissue tropism. The present case lies at the extreme end of the host-vulnerability spectrum: an extremely preterm infant developed bloodstream infection with additional recovery of the organism from urine and BAL. This presentation differs from the early meningitis cases and the prolonged osteoarticular infection reported in an immunocompetent adult but is consistent with the bloodstream and multisite invasive infections described in the Indian series and the predominantly bloodstream phenotype of the Italian pediatric outbreak [27,28,29,30,31,32,33,34]. The two early meningitis cases were fatal [27,28]. Among the three patients identified through Argentinian bloodstream-infection surveillance, one fatal outcome was recorded [30]. The patient with recurrent osteoarticular infection remained clinically stable following repeated surgical source control and prolonged antifungal treatment, including chronic suppressive voriconazole, as well as the two septic arthritis of the knee cases reported in India [32,42]. Of the 21 patients included in the Italian milrinone-associated outbreak, 19 survived; according to the original investigators, the two deaths were related to the patients’ underlying clinical conditions [33,34]. Treatment and outcome data were not reported for the 23 patients in the Indian series [31]. Interestingly, in vivo murine experiments demonstrated relatively low virulence in normal mice but significantly greater morbidity and mortality following cortisone-induced immunosuppression, supporting an opportunistic pathogenic profile [43]. This study, integrated with the few clinical cases, paves the way for further research on *C. viswanathii* virulence factors.

Interpretation of treatment remains considerably more uncertain. Neither species-specific clinical breakpoints nor epidemiological cutoff values are available. The isolates from the present case showed low MICs for amphotericin B and echinocandins, broadly consistent with the other isolates from the Italian outbreak but distinct from the markedly elevated fluconazole MICs reported among a subset of Indian isolates [31,32,33,34,39] (Table S2). Moreover, the infant developed infection despite fluconazole prophylaxis and was subsequently treated with voriconazole followed by the addition of anidulafungin. Although amphotericin B deoxycholate is recommended as first-line treatment for neonatal disseminated candidiasis [44], renal impairment of our patients precludes its use. Intravenous voriconazole (VFEND^®^) was consequently selected as an individualized, off-label bridging treatment, considering its systemic distribution and penetration into the central nervous system [45,46]. Voriconazole is not routinely recommended as initial therapy for neonatal candidiasis, and its pharmacokinetics, optimal dosing and safety in extremely preterm infants remain poorly defined [45,46]. Its use represented a carefully balanced therapeutic compromise. Anidulafungin was subsequently added after identification of *C. viswanathii* and documentation of a low anidulafungin MIC. This provided an additional fungicidal mechanism without requiring renal dose adjustment [44,47,48]. However, this observation cannot establish prophylactic failure or resistance, nor can the efficacy of the therapeutic regimen be assessed, because antifungal exposure was brief and death occurred in the context of profound prematurity, severe cardiorespiratory instability and accidental extubation. In this preterm infant, the fatal outcome cannot be attributed exclusively to candidemia or to any other single cause. It was most likely multifactorial, with extreme prematurity, cardiorespiratory instability, the acute respiratory event and candidemia potentially contributing. More broadly, treatment and outcome data are unavailable for most published patients, preventing meaningful comparisons among antifungal regimens. The current evidence supports isolate-level susceptibility testing but remains insufficient to define an optimal treatment strategy for *C. viswanathii* infection. Moreover, investigation supports extrinsic contamination of a specific milrinone lot, as also highlighted by Ricotta et al. [34], including our case in the first *C. viswanathii* outbreak documented in Europe. Importantly, this cluster occurred against a backdrop of previous, unrelated quality-defect recalls of milrinone infusions, which have repeatedly exposed vulnerabilities in the medication-use pathway.

### 5.2. Evidence Supporting an Iatrogenic Pharmaceutical Source

**Figure 2:**
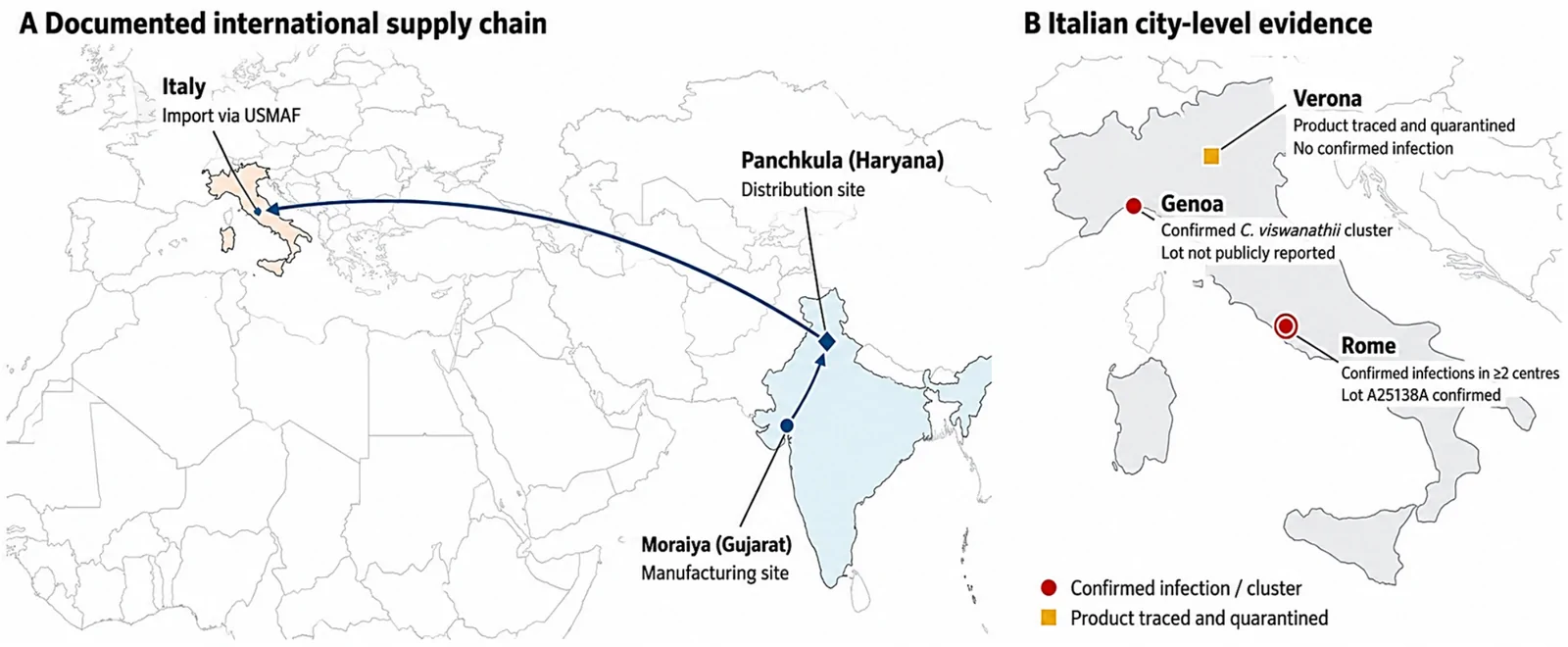
Geographical reconstruction of the documented international pathway and Italian outbreak. USAMF, Uffici di sanità marittima, aerea e di frontiera (Maritime, Aviation and Border Health Offices).

**Figure 3:**
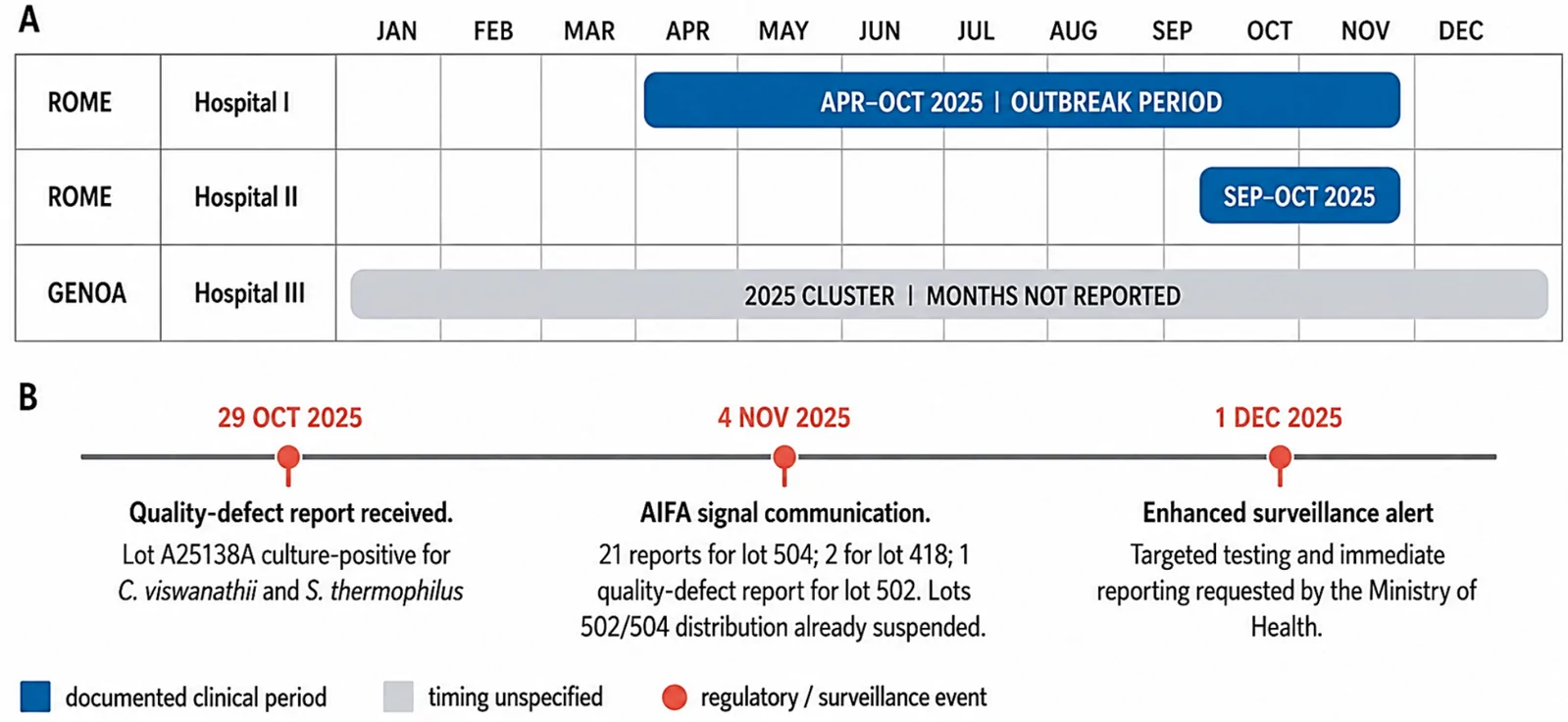
Timeline of documented *Candida viswanathii* clinical reports and pharmacovigilance response in Italy during 2025. (**A**) Center-specific clinical chronology showing the outbreak. Blue bars indicate documented clinical periods, whereas the grey bar denotes unspecified timing. (**B**) Sequence of pharmacovigilance and surveillance events. Orange markers indicate regulatory or surveillance events; their spacing is not proportional to time. The available Italian documents did not specify a formal recall date.

The initial pediatric outbreak described by Vrenna et al. [33] comprised 15 bloodstream isolates recovered from eight patients admitted to intensive-care and cardiology units between April and August 2025. At the time of publication, environmental and patient-screening cultures had not identified a reservoir, and the route of introduction remained unresolved. Later regulatory investigations substantially changed the interpretation of that outbreak. Reports submitted to the Italian Medicines Agency associated *C. viswanathii* infections with milrinone 10 mg/10 mL and involved four lots (502, 504, 418 and A25138A). Lot A25138A, reported by Bambino Gesù Children’s Hospital, yielded *C. viswanathii* and *Streptococcus thermophilus* on culture, providing direct evidence of pharmaceutical contamination [34,40]. Specifically, *C. viswanathii* was recovered in milrinone vials belonging to the same lot administered to our patient (A25138A), analyzed and isolated at the Bambino Gesù Hospital [34]. The Ministry of Health subsequently requested active investigation of milrinone-exposed patients with *Candida*-positive cultures [41]. The Bambino Gesù outbreak was the first iatrogenic cluster associated with contaminated milrinone, even though the medicinal product had not yet been identified as the source when the original microbiological investigation was conducted (Figure 2 and Figure 3). In the subsequent retrospective analysis by Ricotta et al. [34] in the same center, 21 patients (8.5%) developed *C. viswanathii* bloodstream infection, and all had received milrinone before diagnosis. The number of milrinone administrations was the only independent risk factor: patients receiving ≥12 administrations had a more than fivefold higher risk than those receiving fewer administrations, supporting a dose-dependent relationship. No additional cases occurred after withdrawal of the implicated lot. Two infected patients (9.5%) died within 30 days, although both deaths were attributed to their underlying clinical conditions [34].

The present case belongs to the same wider epidemiological event but occurred at a different hospital, involving milrinone lot A25138A as well as for Bambino Gesù [34,39,40,41]. The infant received the product before the onset of candidemia, and *C. viswanathii* was subsequently recovered from two blood cultures, urine and BAL. Moreover, the clinical isolates, previously investigated [39], assigned to *C. viswanathii* from our case grouped with the *C. viswanathii* from the outbreak investigated at the other hospital, which had been processed and characterized by WGS [33,34]. Despite these observations, WGS was not performed on our strains, and genomic relatedness could not be established. In the FTIR analysis, these isolates defined a coherent *C. viswanathii*-associated spectral cluster within the analyzed dataset [39]. Although extreme prematurity, central venous access, parenteral nutrition and broad-spectrum antimicrobial exposure markedly increased the infant’s susceptibility to invasive candidiasis [44,49,50], they do not explain the acquisition of the same uncommon species detected in the medicinal product and among milrinone-exposed patients at another hospital. The pharmaceutical-source hypothesis therefore provides the most coherent explanation for the available observations.

### 5.3. Quality Defects in Parenteral Milrinone

Previous recalls involving milrinone formulations in the United States provide relevant regulatory context and involved different manufacturers, dosage forms and failure mechanisms (Table 2) [51,52] but no microbiological contamination. The 2017 Baxter and 2019 Hospira recalls were prompted by leakage or container defects that compromised sterility assurance and created a potential risk of microbial ingress; neither reported the recovery of a contaminating organism or associated infections. The 2023 Caplin Steriles recall concerned out-of-specification impurities and degradation products. These events demonstrate that milrinone formulations have previously been affected by manufacturing or packaging failures, but they are not microbiological precedents for the 2025 Italian outbreak, in which *C. viswanathii* was detected in a milrinone lot and epidemiologically linked to bloodstream infections among exposed pediatric patients in multiple hospitals in Italy.

### 5.4. Potential Pathways of Exposure

Our case illustrates a complementary scenario in which the yeast most likely entered the NICU via a contaminated intravenous product, instead of through patient-to-patient spread or persistent environmental colonization.

At our institution, source-control and surveillance findings argued against sustained local transmission. After the Bruker Biotyper^®^ and Autof MS2600 (Autobio Diagnostics Co., Ltd., Zhengzhou, China) libraries were updated with spectral data from the seven study isolates, six bloodstream isolates reported as *C. tropicalis* between March and September 2025 were retrospectively re-analyzed; all six were confirmed as *C. tropicalis* [39]. Follow-up blood cultures obtained over the subsequent days from the patient were negative, no additional related cases were detected (another patient has been treated by milrinone lot A25138A, but he has no clinical signs or symptoms related to a candidemia or developed positive cultures), and environmental swabs from monitors, incubators and linens were also negative [39]. Although negative environmental sampling cannot exclude a transient or low-burden reservoir, these findings, together with the absence of new cases after withdrawal of the implicated lot, favor a pharmaceutical source over persistent local environmental transmission [34,39].

From an infection-control perspective, source control in a suspected medication-borne outbreak should therefore extend beyond conventional environmental screening. Immediate quarantine and withdrawal of the implicated lot, preservation and culture of residual or unopened units, retrospective review of phenotypically similar isolates, prospective blood-culture surveillance and interhospital case finding should proceed in parallel [3,21,39]. Together, these observations imply that *C. viswanathii* can reach vulnerable patients through multiple routes and underscore the unresolved question of its persistence outside the clinical setting.

Beyond hospitals, *C. viswanathii* has been recovered from diverse environmental and animal-associated niches, including aquatic habitats, industrial effluents and cockroaches (*Periplaneta americana*) inhabiting hospital sewers [53,54,55,56], placing it at the interface between clinical and environmental settings. This continuum is highly consistent with a One Health framework, in which human infection risk is shaped by interconnected environmental reservoirs, animal-associated vectors and healthcare practices, including the safety of medical products and global supply chains. In this context, preventing rare fungal infections may require integrated surveillance spanning hospitals, surrounding wastewater systems and potential points of contamination along manufacturing and distribution pathways. However, ecological data remain limited and are further complicated by frequent misidentification, so its true reservoirs and routes into healthcare are still uncertain.

### 5.5. C. viswanathii Challenging in Practice

From a diagnostic standpoint, this case underscores how *C. viswanathii* still falls through the gaps of “fast microbiology” workflows. Phenotypic identification systems systematically collapse *C. viswanathii* into *C. tropicalis*, because their assimilation rulesets and databases are optimized for prevalent species within the *Candida*/*Lodderomyces* clade and lack calibrated profiles for rarer taxa [30,57,58,59,60]. In our case, the initial blood culture flagged positive for yeasts, but the BCID2 Panel was negative, reflecting the absence of *C. viswanathii* from its target list [39,60,61,62]. This discordance, in which yeasts are visualized by microscopy, but no corresponding target is detected by the molecular panel, reflects the restricted species coverage of rapid diagnostic panels [21]. In this scenario, MALDI-TOF MS and FTIR provide an important bridge between conventional phenotypic methods and sequencing, but their performances are critically dependent on database curation [39]. In earlier Indian reports, Bruker MALDI-TOF MS using the v3 database returned low-confidence *C. tropicalis* calls for *C. viswanathii* isolates until reference spectra from sequence-proven strains were added [31]. Moreover, Vrenna et al. [33] were also unable to identify *C. viswanathii* by MALDI-TOF. By contrast, in our previous investigation, three independent MALDI-TOF MS systems, equipped with updated libraries that explicitly include *C. viswanathii*, concordantly identified the yeast, and FTIR analyses showed that species genetically related to *C. viswanathii* and *C. tropicalis* are grouped into closely related clusters, albeit distinct from *C. parapsilosis* and *C. auris* [39].

### 5.6. Study Limitations and Practice Recommendations

This report combines clinical, microbiological, pharmaceutical, and regulatory evidence supporting the iatrogenic origin of the infection. However, although our isolates were demonstrated to cluster by FTIR spectroscopy with the WGS-characterized clinical and vial-derived isolates from the Bambino Gesù outbreak [33,34,39], WGS was not performed on our strains, and genomic relatedness could not be conclusively established. The precise point of contamination within the production and distribution chain also remains unknown. Furthermore, the absence of a *post mortem* examination and the complications of extreme prematurity limit assessment of the contribution of candidemia to death. The systematic review is constrained by the small number and heterogeneity of published cases. Overall, this case highlights the importance of lot-level traceability, rapid interhospital communication, and targeted testing of retained units.

## 6. Conclusions

This case extends the Italian milrinone-associated *C. viswanathii* outbreak to an extremely preterm infant. The convergence of clinical infection, documented product exposure, pharmaceutical-quality findings and reports from other centers strongly supports an iatrogenic source, despite the lack of WGS analyses. The central lesson is that unusual, panel-negative yeasts must be connected rapidly to medication-lot traceability. Accurate identification, preservation of isolates and residual products, communication between microbiology and pharmacy, and coordinated pharmacovigilance are all required to recognize and contain pharmaceutical-source fungal outbreaks.

## Figures and Tables

**Table 1:** Published clinical reports of *Candida viswanathii* and the present case. Abbreviations: —, not reported; AFLP, amplified fragment length polymorphism; ANI, anidulafungin; CKD, chronic kidney disease; CSF, cerebrospinal fluid; CVC, central venous catheter; D1/D2, D1/D2 domains of the large-subunit ribosomal RNA gene; F, female; FLC, fluconazole; FTIR, Fourier-transform infrared spectroscopy; ITS, internal transcribed spacer; M, male; MALDI-TOF MS, matrix-assisted laser desorption/ionization time-of-flight mass spectrometry; NICU, neonatal intensive care unit; TDM, therapeutic drug monitoring; TPN, total parenteral nutrition; VRC, voriconazole; WGS, whole-genome sequencing.

Country/Year of Isolation	Clinical Presentation	Sex/Age	Immunity State, Underlying Conditions and Initial Symptoms/Signs	Outcomes/Treatment	Identification	Reference
India, 1959	Meningitis	—/Child	—	*Exitus*	Type strain based on morphology and biochemical characteristics	[27]
India, 1970	Meningitis	M/20 years	Headache	*Exitus*	Morphology and biochemical	[28]
Nigeria, 1987	Asymptomatic vaginal candidiasis	F/—	—	—	Morphology and biochemical	[29]
Argentina, 2005–2009	Bloodstream infection	—/2 pediatric; 1 adult	—	1 *exitus*; 2 survived	Micromorphology, biochemical identification and ITS sequencing	[30]
India, 2013–2015	Invasive candidiasis: blood (12), pus (5), CSF (3), lung nodule/aspirate (2), iliac fluid (1)	16 M, 7 F/—	6/23 neutropenic; chronic disease in 18 patients (tuberculosis, pancreatitis or CKD); 8 post-surgery; 12 with indwelling devices	—	MALDI-TOF MS; D1/D2 sequencing; AFLP	[31]
Singapore, 2020–2025 clinical course	Recurrent septic arthritis and osteomyelitis of the knee	F/78 years	Immunocompetent; osteoarthritis; previous intra-articular lignocaine injections; knee pain and swelling; sinus tract at relapse	Survived; two washouts; 6 months VRC followed by FLC; relapse 14 months after treatment; repeat debridement; long-term VRC	Phenotypic tests; ITS and D1/D2 sequencing	[32]
Italy, 2025	Bloodstream infections	11 M, 10 F/pediatric	Intensive care unit; exposure to contaminated milrinone	2 *exitus*	MALDI-TOF MS; ITS sequencing; FTIR; WGS	[33,34]
Italy, 2025, present case	Candidemia	F/7 days at onset	Preterm infant; birth weight 600 g; NICU admission; CVC, TPN and invasive ventilation; exposure to contaminated milrinone; persistent cardiorespiratory instability	*Exitus*; FLU; VRC; ANI	Three MALDI-TOF MS platforms; ITS sequencing; FTIR	
India, 2026	Subacute septic arthritis of the knee (two cases)	2 M/54 and 75 years	Case 1: Uncontrolled diabetes, hypertension, previous angioplasties and spinal fusion; 3-month knee pain, synovial thickening and joint-line tenderness, without local warmth. Case 2: 3-month knee pain, painful restricted movement and no fever.	Both survived with clinical resolution. Case 1: complete synovectomy and oral FLC for 6 weeks; 18-month follow-up. Case 2: arthroscopic synovial biopsy and oral FLC for 8 weeks; 20-month follow-up.	MALDI-TOF MS	[42]

**Table 2:** Previous recalls involving milrinone formulations. The events differed in manufacturer, formulation and failure mechanism.

Year	Product and Formulation	Quality Defects and Supporting Evidence	Regulatory or Public-Health Response
2017	Premixed milrinone lactate in 5% dextrose, 20 mg/100 mL and 40 mg/200 mL, in single-dose flexible bags.	Customer reports of leaking primary containers, resulting in a lack of assurance of sterility. The action was based on compromised container integrity; no microbial contaminant was reported.	Voluntary FDA Class II recall involving 204,040 bags across two recall records (D-0788-2017 and D-0789-2017) [51].
2019	Milrinone lactate injection in 5% dextrose, 20 mg/100 mL and 40 mg/200 mL, in single-dose flexible bags.	Molding defects affect the additive ports in some units, with potential leakage, microbial ingress, loss of contents or impaired product stability. No associated adverse events had been reported at the time of the recall.	Voluntary recall of 11 lots to the hospital level [52].
2023	Milrinone lactate injection, USP, 20 mg/20 mL (1 mg/mL), in 20 mL single-dose vials.	Failure to meet impurity/degradation specifications during stability testing. This was a chemical-quality defect and did not involve loss of sterility or documented microbial contamination.	Voluntary FDA Class III nationwide recall involving 19,820 vials (D-1144-2023) [51].
2025	Milrinone injection, 10 mg/10 mL. Lots included in the Italian regulatory communications: 502, 504, 418 and A25138A.	Bloodstream infections caused by *C. viswanathii* among exposed pediatric patients. Microbiological testing of lot A25138A yielded *C. viswanathii* and *Streptococcus thermophilus*, providing direct evidence of product contamination [33,34,39,40,41].	Quality-defect and pharmacovigilance notifications to AIFA; national dissemination of the safety signal and active case finding; suspension from clinical use, withdrawal or quarantine of available stock; and coordinated epidemiological and microbiological investigation [33,34,39,40,41].

## Data Availability

The original contributions presented in this study are included in the article. All relevant information is presented in the case report. Further inquiries can be directed at the corresponding authors.

## Supplementary Materials

The following supporting information can be downloaded at: https://www.mdpi.com/article/10.3390/jof12090646/s1. Table S1. MIC values of *C. viswanathii* clinical isolates. AB, amphotericin B; AND, anidulafungin; BAL: bronchoalveolar lavage; CAS, caspofungin; FLZ, fluconazole; ISA, isavuconazole; ITZ, itraconazole; MF, micafungin; POS, posaconazole. * First strain isolated from CVC blood culture. Table S2. In vitro susceptibility of *C. viswanathii* across published studies (1959–2025). All quantitative MICs for the two India cohorts (2013–2014; 2015) come from Shankarnarayan et al. [31]; Morvil et al. [32], Vrenna et al. [33] and De Carolis et al. [39] report CLSI microbroth MIC_50/90_ and ranges for 24 isolates of *C. viswanathii*. PRISMA flowchart file [63]. CARE checklist file.

## Abbreviations

The following abbreviations are used in this manuscript:

BAL	Bronchoalveolar lavage
BCID2	BIOFIRE^®^ Blood Culture Identification 2
CSF	Cerebrospinal fluid
CGMP	Good manufacturing practice
CVC	Central venous catheter
FDA	Food and Drug Administration
FTIR	Fourier-transform infrared
ITS	Internal transcribed spacer
IV	Intravenous
IVH	Intraventricular hemorrhage
MALDI-TOF MS	Matrix-assisted laser desorption/ionization time-of-flight mass spectrometry
MIC	Minimum inhibitory concentration
NICUs	Neonatal intensive care units
SIMV	Synchronized intermittent mandatory ventilation
TPN	Total parenteral nutrition
WGS	Whole-genome sequencing
