# Supplementary material for: The Yeast in the Vial: Iatrogenic Candida viswanathii Infection Associated with Contaminated Milrinone
Source: J Fungi (Basel). 2026 Sep 1;12(9):646. doi: 10.3390/jof12090646 (PMC13608039; doi:10.3390/jof12090646)
Supplement: Supplementary file 1 [file jof-12-00646-s001.zip › CARE checklist.pdf]

CARE
case report guidelines

# CARE Checklist of information to include when writing a case report

(cc) BY-NC-ND

| Topic | Item | Checklist item description | Reported on Line |
|---|---|---|---|
| Title | 1 | The diagnosis or intervention of primary focus followed by the words "case report" | 1-3 |
| Key Words | 2 | 2 to 5 key words that identify diagnoses or interventions in this case report, including "case report" | 44-45 |
| Abstract (no references) | 3a | Introduction: What is unique about this case and what does it add to the scientific literature? | 20-24; 39-42 |
| | 3b | Main symptoms and/or important clinical findings | 28-35 |
| | 3c | The main diagnoses, therapeutic interventions, and outcomes | 28-35 |
| | 3d | Conclusion—What is the main "take-away" lesson(s) from this case? | 39-42 |
| Introduction | 4 | One or two paragraphs summarizing why this case is unique (**may include references**) | 95-110 |
| Patient Information | 5a | De-identified patient specific information. | 113-116 |
| | 5b | Primary concerns and symptoms of the patient. | 115-150 |
| | 5c | Medical, family, and psycho-social history including relevant genetic information | 115-116; family/genetics NR |
| | 5d | Relevant past interventions with outcomes | 118-130 |
| Clinical Findings | 6 | Describe significant physical examination (PE) and important clinical findings. | 113-150 (PE NR) |
| Timeline | 7 | Historical and current information from this episode of care organized as a timeline | 112-166 |
| Diagnostic Assessment | 8a | Diagnostic testing (such as PE, laboratory testing, imaging, surveys). | 117; 126; 131-157 |
| | 8b | Diagnostic challenges (such as access to testing, financial, or cultural) | 131-145; 401-420 |
| | 8c | Diagnosis (including other diagnoses considered) | 131-157; 364-379 |
| | 8d | Prognosis (such as staging in oncology) where applicable | N/A |
| Therapeutic Intervention | 9a | Types of therapeutic intervention (such as pharmacologic, surgical, preventive, self-care) | 118-140 |
| | 9b | Administration of therapeutic intervention (such as dosage, strength, duration) | 118-140; 159-166 |
| | 9c | Changes in therapeutic intervention (with rationale) | 133-140; 274-286 |
| Follow-up and Outcomes | 10a | Clinician and patient-assessed outcomes (if available) | 149-150 (patient N/A) |
| | 10b | Important follow-up diagnostic and other test results | 137-157; 372-376 |
| | 10c | Intervention adherence and tolerability (How was this assessed?) | Adherence N/A; tolerability NR |
| | 10d | Adverse and unanticipated events | 136-150 |
| Discussion | 11a | A scientific discussion of the strengths AND limitations associated with this case report | 423-434 |
| | 11b | Discussion of the relevant medical literature **with references**. | 241-420 |
| | 11c | The scientific rationale for any conclusions (including assessment of possible causes) | 299-335; 364-379 |
| | 11d | The primary "take-away" lessons of this case report (without references) in a one paragraph conclusion | 436-444 |
| Patient Perspective | 12 | The patient should share their perspective in one to two paragraphs on the treatment(s) they received | N/A (neonate; deceased) |
| Informed Consent | 13 | Did the patient give informed consent? Please provide if requested 463-464 | Yes ☑ No ☐ |
