# Supplementary material for: The Yeast in the Vial: Iatrogenic Candida viswanathii Infection Associated with Contaminated Milrinone
Source: J Fungi (Basel). 2026 Sep 1;12(9):646. doi: 10.3390/jof12090646 (PMC13608039; doi:10.3390/jof12090646)
Supplement: Supplementary file 1 [file jof-12-00646-s001.zip › PRISMA 2020 Candida viswanathii.pdf]

PRISMA 2020 flow diagram for new systematic reviews which included searches of databases, registers and other sources

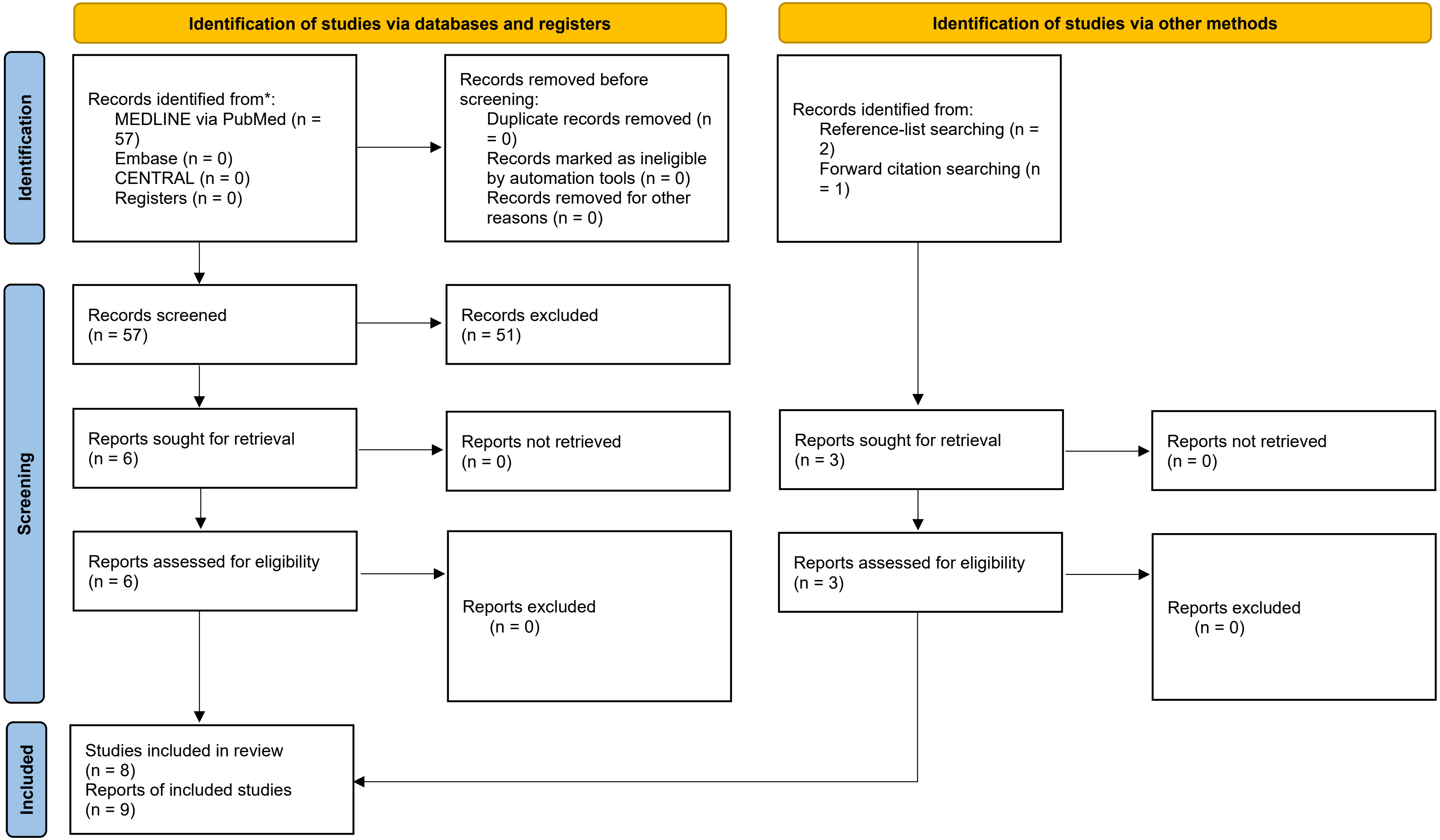

*Consider, if feasible to do so, reporting the number of records identified from each database or register searched (rather than the total number across all databases/registers).
**If automation tools were used, indicate how many records were excluded by a human and how many were excluded by automation tools.

Source: Page MJ, et al. BMJ 2021;372:n71. doi: 10.1136/bmj.n71.

This work is licensed under CC BY 4.0. To view a copy of this license, visit https://creativecommons.org/licenses/by/4.0/
