# Supplementary material for: The Yeast in the Vial: Iatrogenic Candida viswanathii Infection Associated with Contaminated Milrinone
Source: J Fungi (Basel). 2026 Sep 1;12(9):646. doi: 10.3390/jof12090646 (PMC13608039; doi:10.3390/jof12090646)
Supplement: Supplementary file 1 [file jof-12-00646-s001.zip › supplementary files.pdf]

Table S1. MIC values of *C. viswanathii clinical* isolates [39]. AmB, amphotericin B; AND, anidulafungin; BAL: bronchoalveolar lavage; CAS, caspofungin; FLZ, fluconazole; ISA, isavuconazole; ITZ, itraconazole; MF, micafungin; POS, posaconazole. * First strain isolated from CVC blood culture.

Sample ID	Sample Type	AND	MF	CAS	ISA	POS	VOR	ITZ	FLZ	AmB
FPG 2277/25 *	Blood	0.06	0.03	0.12	0.12	0.12	0.12	0.25	2	0.5
FPG 2289/25	Blood	0.06	0.03	0.12	0.12	0.12	0.12	0.25	4	0.5
FPG 2296/25	Urine	0.03	≤0.008	0.06	0.06	0.25	0.12	0.12	4	0.25
FPG 2298/25	BAL	0.03	≤0.008	0.06	0.06	0.25	0.12	0.12	4	≤0.12

Table S2. *In vitro* susceptibility of *C. viswanathii* across published studies (1959–2025). All quantitative MICs for the two India cohorts (2013–2014; 2015) come from Shankarnarayan *et al*. [31]; Morvil *et al.* [32], Vrenna *et al*. [33] and De Carolis *et al.* [39] report CLSI microbroth $MIC_{50/90}$ and ranges for 24 isolates of *C. viswanathii.* NR, not reported.

Antifungal agent	Shankarnarayan *et al*. [31], 2013–2014, n = 7: range; MIC_{50}/MIC_{90}	Shankarnarayan *et al*. [31], 2014–2015, n = 16: range; MIC_{50}/MIC_{90}	Morvil *et al*. [32]	Vrenna *et al*. [33] 2025, n = 15: range	De Carolis *et al* [39]., 2026, n = 7: range
Fluconazole	0.125–1; 1/1	0.5–64; 64/64	4/4	2–4	2–4
Voriconazole	0.03–0.5; 0.5/0.5	0.03–8; 1/2	0.25/0.25	0.25–0.5	0.12
Itraconazole	0.03–0.5; 0.125/0.25	0.03–1; 0.25/0.25	NR	0.12–0.25	0.12–0.25
Posaconazole	0.0312–0.0625; 0.0312/0.0625	0.0312–0.5; 0.25/0.25	NR	0.12–0.25	0.12–0.25
Isavuconazole	NR	NR	NR	0.25–0.5	0.06–0.12
Amphotericin B	0.03–0.25; 0.25/0.25	0.06–0.25; 0.125/0.25	NR	0.25–1	≤0.12–0.5
Caspofungin	0.5; 0.5/0.5	0.125–1; 0.5/0.5	0.12/0.25	0.06–0.25	0.06–0.12
Micafungin	0.125; 0.125/0.125	0.125; 0.125/0.125	NR	0.03–0.12	≤0.008–0.03
Anidulafungin	0.5–1; 0.5/1	0.125–1; 0.5/1	NR	0.06–0.25	0.01–0.06
